# ω3PUFAs improve hepatic steatosis in postnatal overfed rats and HepG2 cells by inhibiting acetyl‐CoA carboxylase

**DOI:** 10.1002/fsn3.2482

**Published:** 2021-07-22

**Authors:** Nan Zhou, Susu Du, Yanyan Dai, Fan Yang, Xiaonan Li

**Affiliations:** ^1^ Department of Child Health Care Children’s Hospital of Nanjing Medical University Nanjing China; ^2^ Institute of Pediatric Research Nanjing Medical University Nanjing China

**Keywords:** ACC, hepatic lipid synthesis, postnatal overfeeding, ω3PUFAs

## Abstract

Postnatal overfeeding can lead to persistent increases in hepatic lipid synthesis and the risk of nonalcoholic fatty liver disease (NAFLD) in adulthood. The ω3 polyunsaturated fatty acids (ω3PUFAs) exhibit beneficial effects on NAFLD. Here, we employed a rat model and an in vitro HepG2 cell model to investigate whether fish oil (FO) affects hepatic lipid synthesis due to postnatal overfeeding. Male Sprague‐Dawley were divided into litter sizes of three (small litters, SLs) or 10 (normal litters, NLs) on postnatal day 3 and were fed standard chow or FO diet beginning on postnatal week 3 to generate NL, SL, NL‐FO, and SL‐FO groups. The results indicated that the FO diet reduced the postnatal overfeeding‐induced body weight gain and NAFLD characteristics (such as serum and liver triglyceride (TG) and hepatic steatosis). In addition, FO restored the expression of hepatic lipid metabolism‐related genes (including *SCD1*, *FASN*, *CPT1*, *LPL*, *ACC*, and *SREBP‐1c*) in SL‐FO rats. Specifically, the activity and expression pattern of ACC were consistent with SREBP‐1c. Furthermore, HepG2 cells were treated with oleic acid (OA), followed by eicosapentenoic acid (EPA), with or without *SREBP‐1c* siRNA. The cellular lipid droplets, TG content, and the expression of ACC (by 75%) and SREBP‐1c (by 45%) were increased by OA stimulation (*p* < .05), which was inhibited by EPA treatment. However, the effect of EPA treatment was abolished when SREBP‐1c was silenced. In conclusion, ω3PUFAs‐rich diet may be an effective way to reverse the developmental programming of hepatic lipid synthesis, at least partially, by inhibiting ACC through modulating SREBP‐1c.

## Graphical abstract

Post‐weaning fish oil diet could improve hepatic steatosis and decrease the risk of developing NAFLD in later life, which emphasizing that the ω3PUFAs supplementation should be encouraged in obese children, especially those who have experienced postnatal overfed.

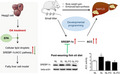

## 1 INTRODUCTION

With the increasing prevalence of obese children, nonalcoholic fatty liver disease (NAFLD) has become one of the most common chronic liver diseases in pediatric population (Flisiak‐Jackiewicz & Lebensztejn, 2019; Hales et al., 2017). The overall prevalence of pediatric NAFLD is approximately 10%, including up to 17% in teenagers and 40%–70% among obese children (Clemente et al., 2016). The onset of pediatric NAFLD is often asymptomatic, and hepatic lipid accumulation in the early period of NAFLD could more easily lead to significant hepatic and systemic consequences, including nonalcoholic steatohepatitis (NASH) and cirrhosis (Fang et al., 2018; Nobili et al., 2016). Therefore, it is urgent need to identify characteristics of hepatic lipid accumulation in early life to prevent of pediatric NAFLD progress.

Infant growth trajectory is a major determinant of metabolic health later in life (Parra‐Vargas et al., 2020). Experimental evidence has indicated that overnutritional environment in critical windows (fetal, infant to adolescent) can reset metabolic pathways by physiologic functional and structural changes and has permanent effects throughout adulthood; this phenomenon is a key feature of developmental programming(Barker & Osmond, 1986; Körner et al., 2019; Ratnasingham et al., 2017; Roseboom et al., 2006). Small litter (SL) rearing is a classic animal model that has been used to simulate overnutritional environment in postnatal early life (Habbout et al., 2013; Hou et al., 2011; Mozes et al., 2014; Yzydorczyk et al., 2019). SL rats and mice exhibit rapid weight gain and early‐onset of obesity, hepatic steatosis, and other tissue metabolic defects (Preston et al., 2018; Ramon‐Krauel et al., 2018). Consistent with these reports, our previous experiment have also shown that SL rats promoted the early‐onset and exaggerated the obesity and NAFLD induced by high‐fat diet (Ji et al., 2014), whereas rats reared in normal litters (NL) fully or partially reversed to normal weight, even after developing obesity from the initial high‐fat diet (Hariri & Thibault, 2010). Therefore, postnatal early life is considered to be critical window of metabolic disorders, and further exploration of effective preventive strategies is important.

NAFLD is characterized by excessive triglyceride (TG) accumulation in the absence of significant alcohol consumption (Aguila, 2011) and is primarily caused by the imbalance in which lipid availability (uptake and synthesis) exceeds lipid disposal (oxidation and export; Kjaergaard et al., 2014; Musso et al., 2009). It is well‐known that hepatic lipid metabolism, including circulating lipid uptake, de novo lipogenesis (DNL), fatty acid oxidation, and TG‐rich lipoprotein secretion, is regulated by several rate‐limiting enzymes and transcription factors(Kjaergaard et al., 2014). Specifically, acetyl‐CoA carboxylase (ACC), stearyl coenzyme A saturated enzyme 1 (SCD1), and fatty acid synthase (FASN) are crucial in DNL (Kim, 1997). In addition, liver‐type fatty acid‐binding protein (L‐FABP) and lipoprotein lipase (LPL) are mainly responsible for fatty acid intake, and carnitine palmitoyltransferase 1 (CPT1) and microsomal triglyceride transfer protein (MTP) are involved in oxidation and export (White et al., 1998). Moreover, the activities of these enzymes are regulated by transcription factors, such as sterol regulatory element‐binding protein‐1c (SREBP‐1c) and peroxisome proliferator‐activated receptor α (PPARα; Andrews et al., 2008; Spann et al., 2006). In our previous study, we found that postnatal overfed rats permanently increased hepatic ACC and SREBP‐1c expression from weaning to adulthood even with the consumption of a postweaning standard diet. However, CPT1 and PPARα expressions decreased when insulin resistance occurred (Ji et al., 2014). All such observations indicate that the overnutritional in early life can affect the expression of those enzymes involved in hepatic lipid synthesis and reprogram such metabolic pathways in later life.

The ω3 polyunsaturated fatty acids (ω3PUFAs), which are particularly rich in fish oil (FO), can reduce hepatic steatosis and increased anti‐oxidative capacity, which exhibits effectiveness in alleviating NAFLD in adults and children (Parker et al., 2012), while ω3PUFAs‐deficient diets were closely associated with greater portal and lobular inflammation in NAFLD patients (St‐Jules et al., 2013). According to the Dohad theory and our previous experiments, postnatal overfeeding could affect hepatic lipid metabolism earlier and heavier, and these programmatic changes are not easily affected by standard diets in later life. Therefore, it is needed to confirm if ω3PUFAs can improve hepatic metabolic disorders induced by postnatal overfeeding.

Herein, we hypothesized that ω3PUFAs diet after weaning could reverse the developmental programming of hepatic lipid metabolism and prevent the development of the fatty hepatic steatosis in postnatal overfed rats. This study focused specifically on the effects of ω3PUFAs on hepatic lipid synthesis and the underlying molecular mechanisms in early life.

## 2 MATERIALS AND METHODS

### 2.1 Animals and experimental design

**FIGURE 1:**
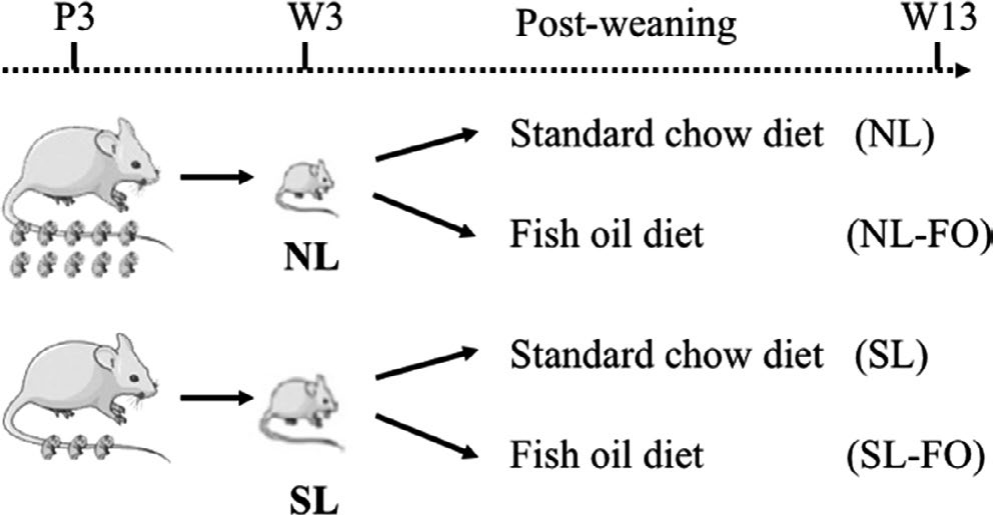
Experimental protocol. FO, fish oil; NL, normal litters; P3, postnatal day 3; SL, small litters; W13, postnatal week 13; W3, postnatal week 3

The protocols employed in this study were approved by Unit for Laboratory Animal Medicine at Nanjing Medical University (ID:20130102‐01). The experimental setup was similar to that described in Boullu‐Ciocca (Boullu‐Ciocca et al., 2008). For rats, pups were weaned at postnatal week 3, puberty was reached at postnatal weeks 6–8, and adulthood was attained at postnatal week 9. According to our previous studies, we chose weeks 3 and 13 as experimental time points to examine the effects of early nutrition on adult health (Ji et al., 2014). At postnatal day 3 (P3), male pups were divided into litter sizes of three (small litters, SL) or ten (normal litters, NL) to induce early postnatal overfeeding or normal feeding, respectively (Rodrigues et al., 2009; Velkoska et al., 2008). After weaning (P21, W3), rats were fed either a chow diet or a FO diet rich in ω3PUFAs. The dietary nutrient compositions are shown in Table 1, and the fatty acid compositions of the diets are shown in Table 2 (Slac, Shanghai, China). Water intake was optional until postnatal week 13 (W13). Four groups were named as NL, SL, NL‐FO, and SL‐FO (Figure 1). There were nine rats in each group and were housed three per cage after W3. Body weights and food intakes were monitored throughout life. Room temperature was maintained at 22℃, and light was maintained on a 12:12‐hr light–dark cycle. At the end of the experiments, rats were euthanased. Serum and liver samples were collected and stored at −80℃ for further use.

**TABLE 1:** Purified diet formula and composition [weight (%)]

	Chow diet (%)	6%ω3PUFAs (%)
Casein	18.92	18.92
L‐Cystine	0.28	0.28
Corn starch	48.34	48.34
Maltodextrin	3.32	3.32
Sucrose	13.00	13.00
Cellulose	4.74	4.74
Soybean oil	6.00	—
Fish oil	—	6.00
Mineral mix	4.26	4.26
Vitamin mix	1.14	1.14
Total	100.00	100.00
Energy (kcal/100 g)	392.60	392.60

**TABLE 2:** Fatty acid profile of the diets (mg/100 mg)

	Chow diet	ω3PUFAs
LA (C18:2)	1.437	0.806
AA (C20:4)	0.003	0.013
Total *n*‐6PUFA	1.437	0.819
ALA (C18:3)	0.135	0.078
EPA (C20:5)	0.041	0.343
DPA (22:5)	0.004	0.035
DHA (C22:6)	0.028	0.561
Total *n*‐3 PUFA	0.207	1.017
Total *n*‐3/*n*‐6 PUFA	0.144	1.242

Abbreviations: AA, Arachidonic acid; ALA, Linolenic acid; DPA,DHA, Do cosahexaenoic acid; EPA, Eicosapentaenoic; LA, Linoleic acid; PUFA, polyunsaturated fatty acid.

### 2.2 Intraperitoneal glucose tolerance test (IPGTT)

IPGTT was performed as described previously (Chen et al., 2008). Briefly, rats were fasted overnight at W3 and W13, blood samples were obtained from the tail vein, and 2.0 g of D‐glucose (50% stock solution in saline)/kg of body weight was injected intraperitoneally. Subsequently, blood samples were obtained from the tail vein at 30‐, 60‐, 90‐, and 120‐min intervals following glucose injection. Plasma glucose levels were measured using an Accu‐Chek Performa glucometer. Total AUC was determined here.

### 2.3 Serum biochemistry and hepatic lipid assay

Total triglyceride (TG), total cholesterol (TC), and high‐density lipoprotein (HDL) in serum were measured using enzymatic colorimetric assays on an Olympus AU400 analyzer. The colorimetric assays were performed using TCHOD‐PAP reagent kit 20090 and GPO‐PAP reagent kit 20080 (BIOSINO BIO), according to the manufacturer's instructions. Hepatic TG concentrations were expressed with 1 g of liver protein correction. The hepatic protein concentrations were measured by a modified Folch extraction method, as described previously (Obrowsky et al., 2013), using a Pierce bicinchoninic acid (BCA) protein assay kit and bovine serum albumin (BSA) as the standard (Thermo Fisher Scientific).

### 2.4 Hepatosomatic index

Whole livers were removed from rats, washed with ice‐cold normal saline, blotted dry, and weighed. The hepatosomatic index was calculated as follows: (liver weight/body weight) × 100% (Al‐Ghais, 2013).

### 2.5 Hepatic histological analyses

Portions of the left and right lobes were either flash‐frozen in isopentane (Oil Red O) or fixed in buffered formalin [hematoxylin and eosin (H&E)] for histological analyses.

### 2.6 NAFLD activity score (NAS)

Based on the study by Kleiner et al. (2005), the NAFLD activity score was generated by the sum of the individual scores of the following parameters: steatosis (<5% = 0; 5%–33% = 1; 33%–66% = 2; >66% = 3); lobular inflammation (none = 0; <2 foci = 1; 2–4 foci = 2; >4 foci = 3); ballooning (none = 0; few = 1; prominent = 2). A score of <3 correlates with mild nonalcoholic fatty liver, while a score of 3–4 correlates with moderate nonalcoholic fatty liver, and a score ≥5 correlated with NASH. The average score for each histological characteristic in each group (*n* = 6) was utilized. A minimum of three slices per liver tissue sample, and ten fields of vision each slide were evaluated to determine the NAS.

### 2.7 Cell cultures and treatments

Hepatocellular carcinoma cell line (HepG2 cells) was cultured in high‐glucose Dulbecco's modified Eagle's medium (DMEM) supplemented with 10% fetal bovine serum (FBS) and penicillin (100 IU/ml)–streptomycin (100 μg/ml). Oleic acid (OA) was used as a steatosis vector for HepG2 cells. After reaching 80% confluency, cells were exposed to 2.0 mmol/L OA and 1% FFA‐free BSA in DMEM for 24 hr. Then, the medium was replaced by a mixture including 1% free fatty acid (FFA‐free) BSA and 100 µm/L eicosapentenoic acid (EPA) in DMEM for 24 hr. All the cells were incubated at 37℃ in a humidified atmosphere with 5% CO_2_. Total RNA and total protein were extracted from harvested cells for mRNA and protein expression using transcription quantitative polymerase chain reaction (RT‐qPCR) and Western blotting, respectively.

### 2.8 RNA interference

The specific siRNA against SREBP‐1 was synthesized by GenePharma Co., Ltd.. At 70%–80% confluency, HepG2 cells were transfected with siRNA‐SREBP‐1c using siRNA Transfection Reagent, according to the manufacturer's instructions. The transfection efficiency was measured by counting the number of EGFP‐positive cells under a fluorescence microscope. The medium was replaced after transfection for 4–6 hr post‐transfection. After 24 hr, the cells were treated with OA for 24 hr and EPA for an additional 24 hr. Nontargeting siRNA was used as a negative control.

### 2.9 RT‐qPCR

Total RNA was extracted from hepatic tissues and HepG2 cells using TRIzol reagent (Thermo Fisher Scientific, Inc.). The integrity of total RNA was assessed using agarose gel electrophoresis, and cDNA was synthesized using M‐MLV reverse transcriptase (Promega Corporation) with 1.0 μg of the RNA sample, according to the manufacturer. Genes of interest were analyzed by real‐time PCR using SYBR GREEN and an ABI Prism 7500 Real‐Time PCR System for the target genes *ACC*, *FASN*, *SCD1*, *L‐FABP*, *LPL*, *CPT1*, *MTP*, and *SREBP‐1c*. The relative gene expression was normalized to that of glyceraldehyde‐3‐phosphate dehydrogenase (*GAPDH*) using the 2^−ΔΔct^ method (Pfaffl, 2001). The primers are listed in Table 3.

**TABLE 3:** Primer sequences used for mRNA quantification by real‐time PCR

	Forward primer 5′‐3′	Reverse primer 5′‐3′
ACC rat	TGAAGGGCTACCTCTAATG	TCACAACCCAAGAACCAC
LPL rat	GCTTCCCCTTACTGGTTCC	AACTGGCAGGCAATGAGACT
SREBP‐1c rat	CGCTACCGTTCCTCTATCA	CTCCTCCACTGCCACAAG
CPT1 rat	ACGAAGAACATTGTGAGCGG	GAGGACCTTGACCATAGCCA
MTP rat	AGCAACATGCCTACTTCTTACAC	TCACGGGTTCACTTTCACTG
FASN rat	AAGAGTGGGAGAGCCTGTTC	AGTTCACCAAGCCTACCACA
SCD1 rat	CTCCCTACCTCCACCCCTAT	AACCAACCCTCTCGTTCAGT
L‐FABP rat	AAGGGAAGGACATCAAGGGG	CACTGCCTTGACCTTTTCCC
GAPDH rat	GGCTCTCTGCTCCTCCCTGTTCTA	CGTCCGATACGGCCAAATCCGT
ACC HepG2	CACGCTCAAGTCACCAAGAA	GCAAATGGGAGGCAATAAGA
SREBP‐1c HepG2	TTCCCAGCCCCTCAGATAC	GAGAAGCACCAAGGAGACGA
GAPDH HepG2	GTCGGAGTCAACGGATTTGG	CATGGGTGGAATCATATTGGA

Abbreviations: ACC, acetyl‐CoA carboxylase; CPT1, carnitine palmitoyltransferase1; FASN, fatty acid synthase; GAPDH, glyceraldehyde‐3‐phosophate dehydrogenase; L‐FABP, liver‐type fatty acid‐binding protein; LPL, lipoprotein lipase; MTP, microsomal triglyceride transfer protein; SCD1, stearyl coenzyme A saturated enzyme 1; SREBP‐1c, sterol regulatory element‐binding protein‐1c.

### 2.10 Protein extraction and Western blot analysis

Rat liver tissues were homogenized in ice‐cold radio immunoprecipitation assay (RIPA) buffer. Then, the cells were harvested and lysed in a CelLytic™ MT cell lysis reagent (Sigma‐Aldrich) for protein extraction. Protein concentration was determined using a Pierce BCA protein assay kit with BSA as the standard (Thermo Fisher Scientific). An equivalent of 40 mg protein per sample was separated by 10% sodium dodecyl sulfate‐polyacrylamide gel electrophoresis (SDS‐PAGE) and transferred onto nitrocellulose filter membranes (0.45 mm; Millipore) at 4℃. Then, the membranes were blocked in 5% nonfat milk for 1.5 hr at room temperature and probed with a primary antibody against SREBP‐1c (Abcam), or β‐actin (Santa Cruz) at 1:1,000 dilution overnight at 4℃. Subsequently, the membranes were incubated with the second antibodies for 1 hr; the immune‐reactive bands were visualized using chemiluminescence, and the intensity was analyzed using a Gel‐Pro analyzer 4.0 software.

### 2.11 ACC activity assay

The assay for ACC activity in liver was based on the acetyl‐CoA‐dependent formation of acid‐stable radioactivity derived from H^14^CO_3_^−^(Alberts & Vagelos, 1968). The homogenates of rat livers were diluted 1:4 ratio with KCL, and the supernatants were collected by centrifugation at 9,000 *g* at 4℃ for 20 min. The supernatant was prepared to assess the enzymes content by the ACC assay, following the method of Craig et al. (Inoue & Lowenstein, 1975). The activity was expressed in mU/mg of protein, and the protein content was determined by the Lowry’ method (Lowry et al., 1951).

### 2.12 Statistical analysis

The data were expressed as mean ± *SEM*. Repeated‐measures analysis of variance (ANOVA) was used to analyze the body weight gain and energy intake of rats. The two‐sided Student's *t* test was used to detect significant differences between NL and SL rats at week 3. Significant differences among groups of rats were analyzed by one‐way analysis of variance (ANOVA), followed by the least significant difference (LSD) test. The effects of different treatments in HepG2 culture were analyzed by multi‐factor analysis of variance. A *p*‐value <0.05 was considered statistically significant.

## 3 RESULTS

### 3.1 Body weights, food, and energy intake

**FIGURE 2:**
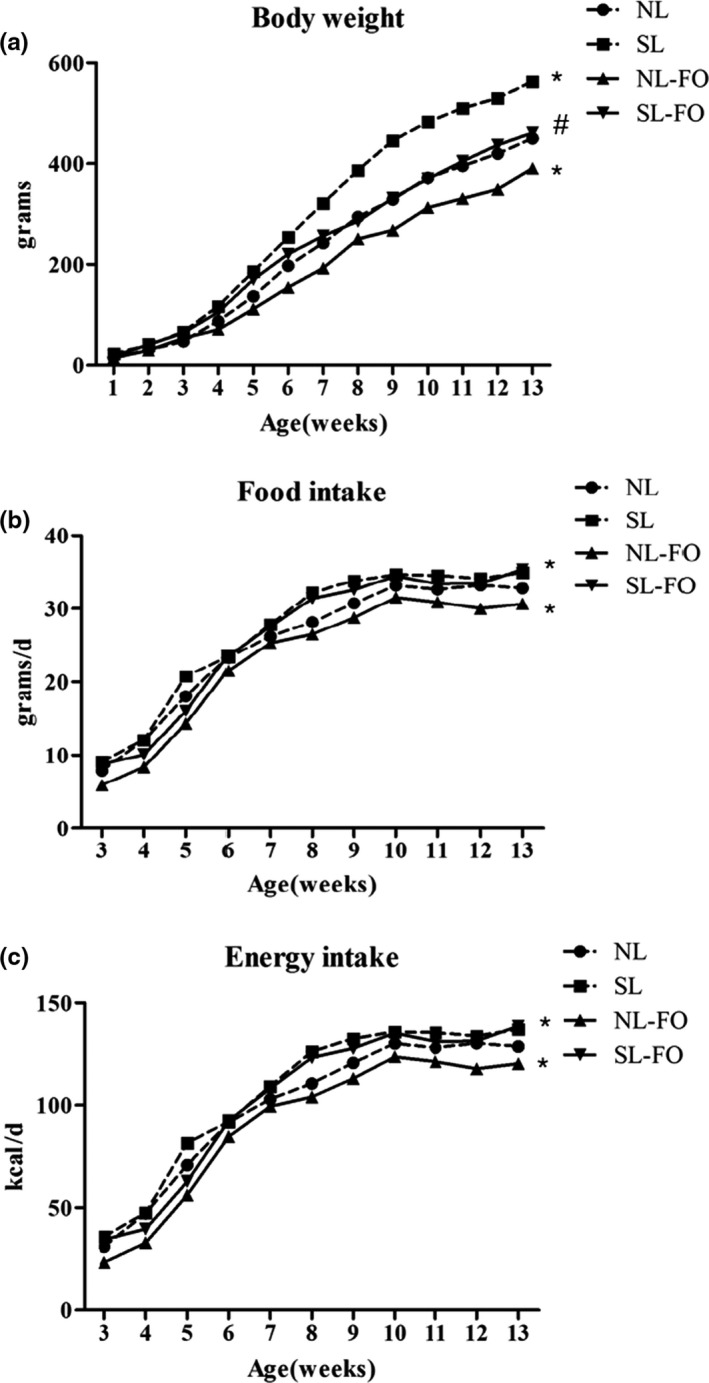
Phenotypic characteristics of rats. (A) Body weight, and (B) food and (C) energy intake in rats from W3 to W13. *p < .05 versus NL rats. ^#^ *p* < .05 versus SL rats. The data are expressed as the mean ± *SEM*. *n* = 9 per group. FO, fish oil; NL, normal litters; SL, small litters; W, postnatal week

From as early as W3, and all the way through to W13, SL rats displayed higher body weights, food, and energy intake (*p* < .05; Figure 2A–C) than NL rats. On the other hand, SL‐FO rats had lower food intake than those of SL rats from W3 to W5, but from W6 they started to approach the level of food intake of the SL rats. By W13, no difference in food intake was observed between SL‐FO and SL rats. However, the body weights of SL‐FO rats were lower than those of SL rats from W6 to W13 (*p* < .05; Figure 2A–C). The NL‐FO rats exhibited lower food intakes from W3 onwards and lower body weights from W4 onwards (*p* < .05; Figure 2A–C), both of which persisted until W13, as compared to NL rats.

### 3.2 Serum lipid and glucose homeostasis

As shown in Table 4, higher serum TG contents were detected in SL rats compared to NL rats (*p* < .05), while it was decreased in SL‐FO rats compared to SL rats (*p* < .05). No significant differences were detected in TC contents among the four groups. However, the HDL content was higher in SL‐FO rats than that in SL rats (*p* < .05).

**TABLE 4:** Serum lipid parameters in rats at W13 (mean ± *SEM*)

	NL	SL	NL‐FO	SL‐FO
TG (mmol/L)	0.40 ± 0.05	0.77 ± 0.10*	0.38 ± 0.06	0.42 ± 0.39^#^
TC (mmol/L)	1.23 ± 0.09	1.35 ± 0.15	1.26 ± 0.21	1.40 ± 0.07
HDL (mmol/L)	0.46 ± 0.01	0.51 ± 0.04	0.41 ± 0.05	0.61 ± 0.04^#^

Abbreviations: HDL, high‐density lipoprotein cholesterol; TC, cholesterol; TG, triglyceride, *n* = 9 in each group.

* *p* < .05 versus NL.

^#^ *p* < .05 versus SL.

**FIGURE 3:**
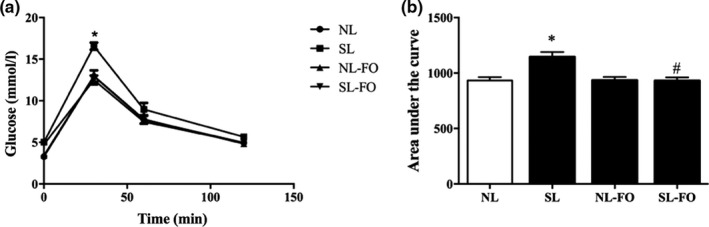
Intraperitoneal glucose tolerance test and area under the curve analyses in rats at W13. *p < .05 versus NL rats. ^#^ *p* < .05 versus SL rats. The data are expressed as the mean ± *SEM*. *n* = 9 per group. FO, fish oil; NL, normal litters; SL, small litters. W13, postnatal week 13

As shown in Figure 3, the AUC for plasma glucose was highest in SL rats (*p* < .05), while plasma glucose levels were decreased in SL‐FO rats (*p* < .05) at W13, and no significant difference was observed in plasma glucose levels between NL and NL‐FO rats.

### 3.3 Liver weight and hepatic TG content

As shown in Table 5, liver weights and hepatic TG contents in SL rats were higher than those of NL rats from W3 to W13 (*p* < .05). The liver weights and hepatic TG contents in SL‐FO rats were decreased compared to SL rats (*p* < .05). Similarly, liver weights and TG contents in NL‐FO rats were lower than those in NL rats (*p* < .05).

**TABLE 5:** Hepatic phenotypic characterization in rats at W3 and W13 (mean ± *SEM*)

	NL	SL	NL‐FO	SL‐FO
W3				
Liver weight (g)	1.23 ± 0.18	3.23 ± 0.23*	—	—
HIS (%)	2.31 ± 0.31	4.52 ± 0.28*	—	—
Liver TG (mmol/g)	0.12 ± 0.01	0.13 ± 0.08	—	—
W13				
Liver weight (g)	13.23 ± 0.38	15.95 ± 0.58*	11.26 ± 0.21*	13.95 ± 0.63^#^
HIS (%)	2.83 ± 0.06	3.15 ± 0.07*	3.01 ± 0.05	3.09 ± 0.04
Liver TG (mmol/g)	0.12 ± 0.006	0.15 ± 0.004*	0.11 ± 0.006	0.13 ± 0.005^#^

Note

The hepatosomatic index (HIS) was calculated as follows: (liver weight/body weight) × 100%. TG, triglyceride, *n* = 9 per group.

* *p* < .05 versus NL.

^#^ *p* < .05 versus SL.

### 3.4 Hepatic histological examination

**FIGURE 4:**
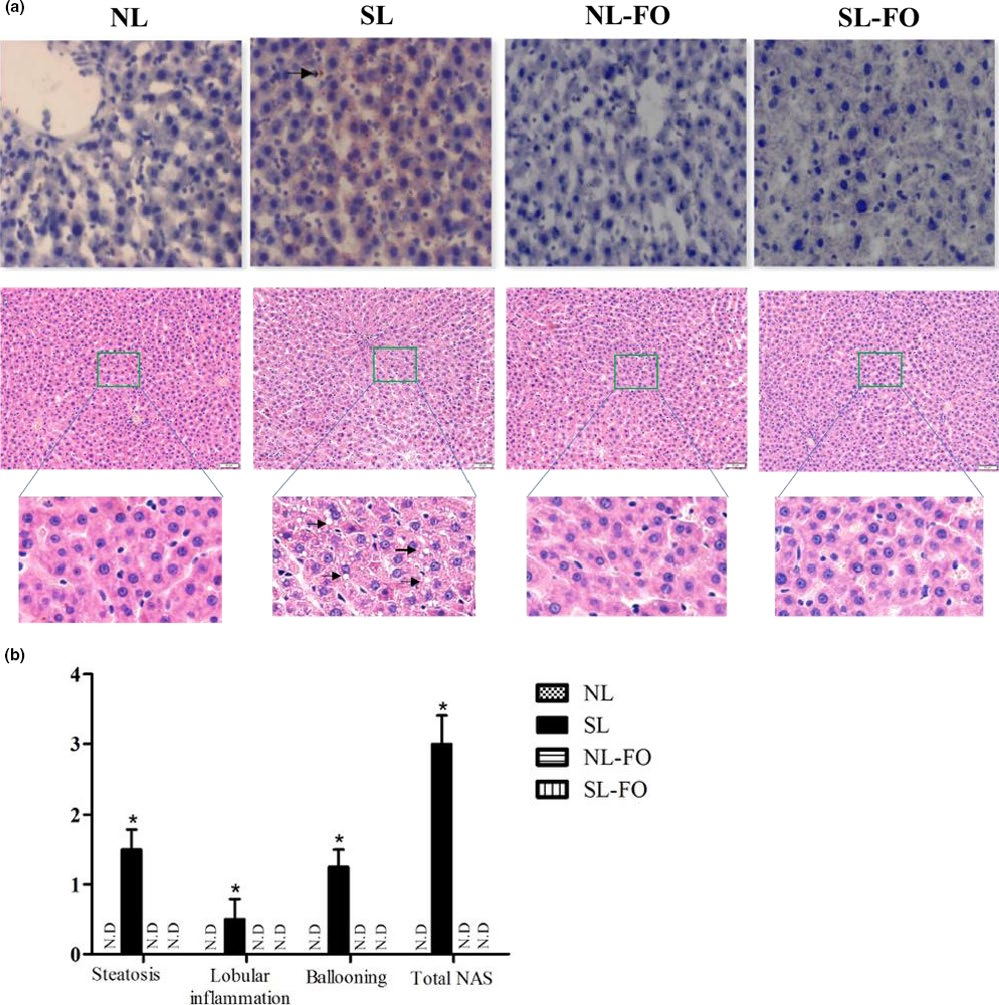
(A) Histological analysis and (B) NAS in rats at week 13. SL rats exhibited moderate steatosis (large arrow) and hepatocyte ballooning (arrow head; H&E staining) with a greater degree of steatosis (Oil Red O staining). Magnification for light microscopy, ×200. *SL rats achieved a NAS of 2.8, which was higher than that of other groups (*p* < .05). The data are expressed as the mean ± *SEM*. *n* = 9 per group. NAS, nonalcoholic fatty liver disease activity score; H&E, hematoxylin and eosin; FO, fish oil; N.D, no droplets; NL, normal litters; SL, small litters

As shown in Figure 4, SL rats exhibited hepatic steatosis (H&E), which was also confirmed by Oil Red O staining. The livers of SL rats were filled with small droplets and accompanied by ballooning in hepatocytes. Conversely, no droplets and ballooning were observed in the livers of the other groups (Figure 4A). The severity of the NAFLD was assessed using NAS. SL rats achieved a higher NAS (2.8) than other groups, which indicated hepatic steatosis (*p* < .05; Figure 4B).

### 3.5 mRNA expression of rate‐limiting enzymes in hepatic tissue

**FIGURE 5:**
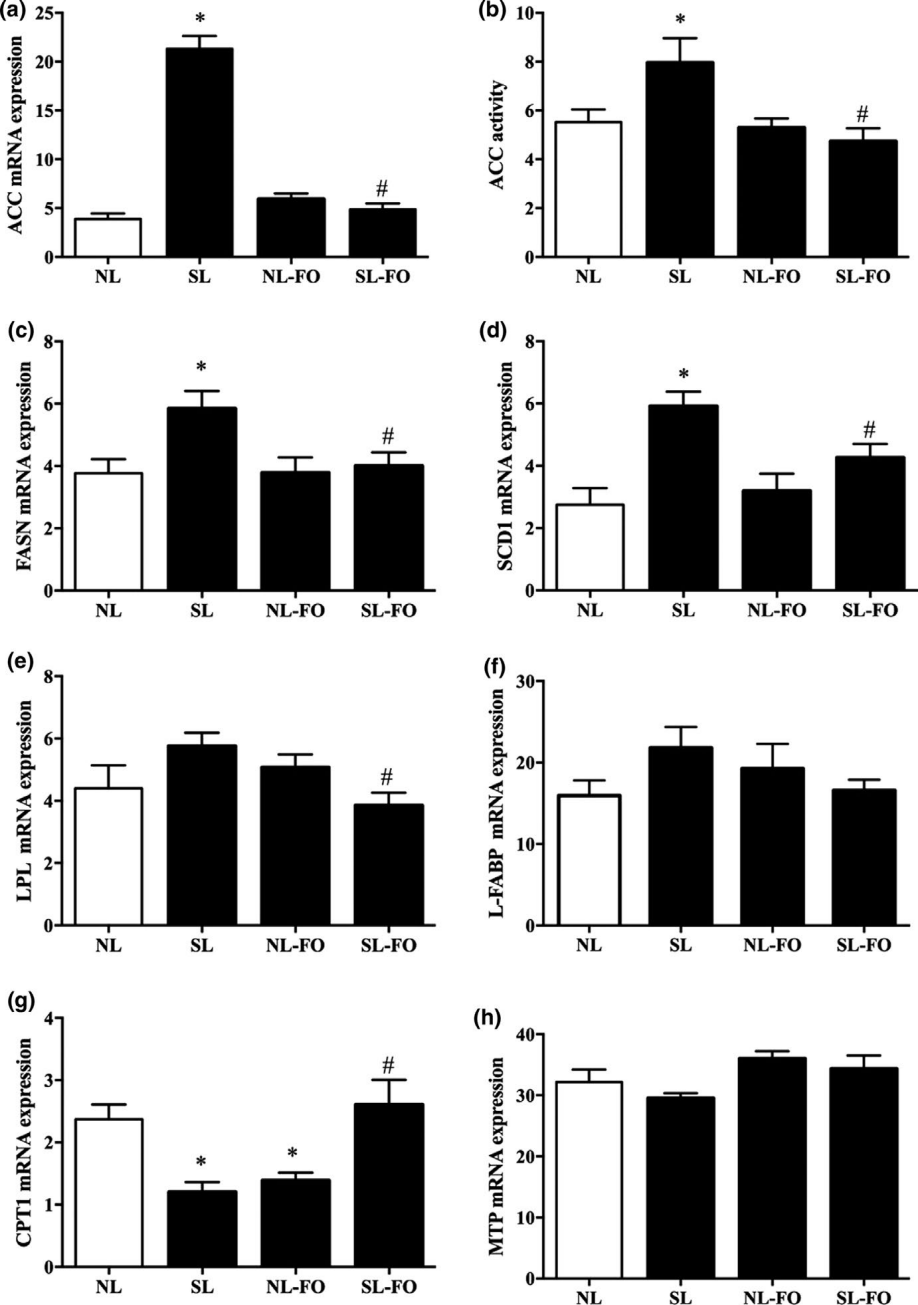
Effects of the FO diets on the enzymes involved in hepatic lipid metabolism at week 13. The enzyme activity of (B) ACC and the mRNA expression of (A) ACC, (C) FASN, (D) SCD1, (E) LPL, (F) L‐FABP, (G) CTP1, and (H) MTP. *p < .05 versus NL rats. ^#^ *p* < .05 versus SL rats. The data are expressed as the mean ± *SEM*. *n* = 9 per group. ACC, acetyl‐CoA carboxylase; CTP1, carnitine palmitoyl transferase 1; FASN, fatty acid synthase; L‐FABP, liver‐type fatty acid‐binding protein; LPL, lipoprotein lipase; MTP, triglyceride transfer protein; SCD1, stearyl coenzyme A saturated enzyme 1

Of note, the ACC mRNA expression was increased by fivefold in SL rats, as compared to that in NL rats, but was decreased by FO diet at W13 (*p* < .05; Figure 5A). However, ACC mRNA expression in NL rats did not differ from that in NL‐FO rats. The enzyme activity of ACC was consistent with the mRNA expression (Figure 5B). Moreover, the SCD1 and FASN expressions were increased in SL rats but reduced in SL‐FO rats (*p* < .05; Figure 5C,D). The levels of CPT1 were markedly decreased in SL rats, while CPT1 expression was enhanced by FO diet (*p* < .05; Figure 5G). A lower LPL expression was observed in SL‐FO rats, as compared to that in SL rats (Figure 5E). Of note, no significant differences were observed in the L‐FABP and MTP expression in any of the four groups (Figure 5F,H).

### 3.6 SREBP‐1c expression in hepatic tissue

**FIGURE 6:**
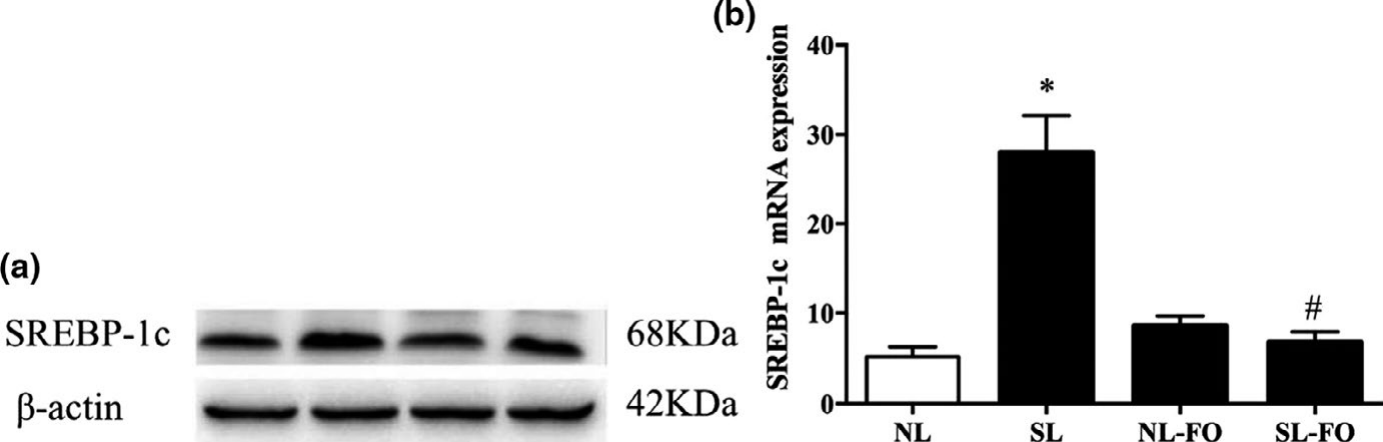
SREBP‐1c mRNA and protein expression in hepatic tissue. *p < .05 versus NL rats. ^#^ *p* < .05 versus SL rats. The data are expressed as the mean ± *SEM*. *n* = 9 per group. NL, normal litters; SL, small litters; SREBP‐1c, sterol regulatory element‐binding protein‐c

As shown in Figure 6, the SREBP‐1c mRNA and protein expression was significantly increased in SL rats than NL rats. However, the FO diet rearing attenuated SREBP‐1c expression at W13 (*p* < .05), which was consistent with the ACC expression pattern.

### 3.7 Effects of SREBP‐1c siRNA interference

**FIGURE 7:**
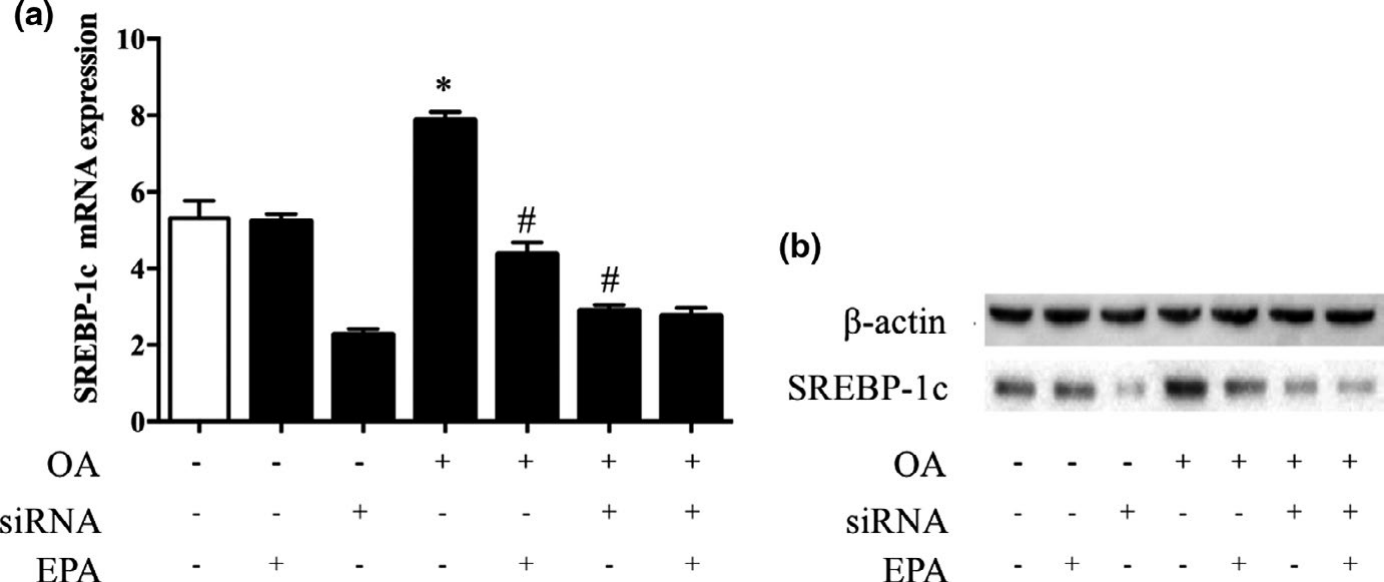
Effects of SREBP‐1c siRNA interference. (A) SREBP‐1c mRNA and (B) protein expression in HepG2 cells with different treatments: (i) Control, (ii) EPA, (iii) SREBP‐1c siRNA, (iv) OA, (v) OA+EPA, (vi) SREBP‐1c siRNA+OA, and (vii) SREBP‐1c siRNA+OA+EPA. *p < .05 versus controls, ^#^ *p* < .05 versus OA treatment alone. The data are expressed as the mean ± *SEM*. *n* = 6 in each treatment. EPA, eicosapentaenoic acid; OA, oleic acid; SREBP‐1c, sterol regulatory element‐binding protein‐c

As shown in Figure 7, OA increased the expression of SREBP‐1c, while EPA significantly decreased its expression in the absence of siRNA interference (*p* < .05; Figure 7A). When SREBP‐1c was blocked by siRNA interference, the SREBP‐1c expression was successfully reduced by about ~60% (*p* < .05; Figure 7A). Strikingly, OA did not increase the expression of SREBP‐1c, and EPA failed to down‐regulate SREBP‐1c. Additionally, the mRNA expression of SREBP‐1c was consistent with its protein expression (Figure 7B).

### 3.8 Lipid accumulation in HepG2 cells

**FIGURE 8:**
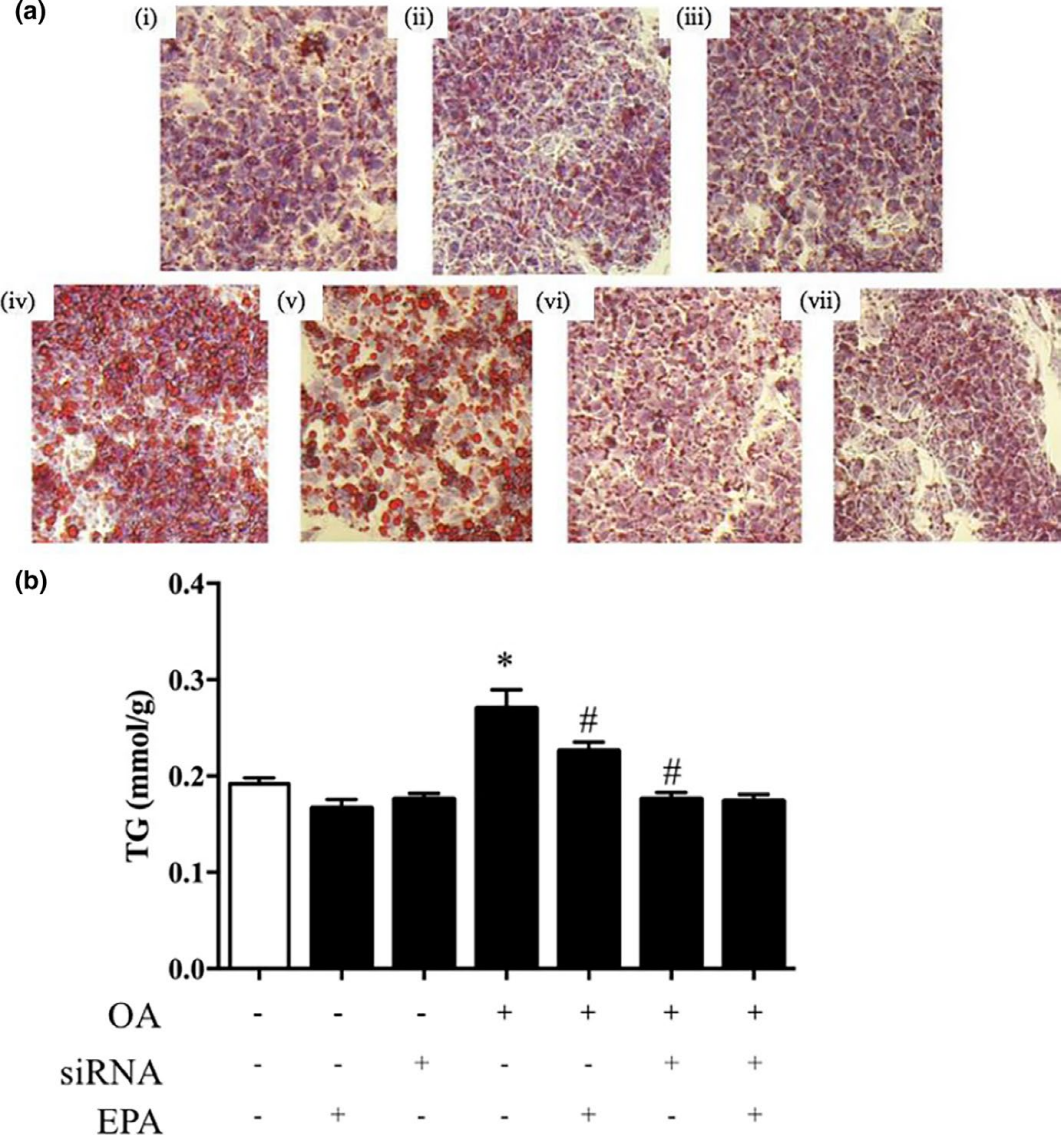
EPA attenuates lipid accumulation in HepG2 cells. (A) Oil red staining and (B) TG content of HepG2 cells under different treatments: (i) Control, (ii) EPA, (iii) SREBP‐1c siRNA, (iv) OA, (v) OA+EPA, (vi) SREBP‐1c siRNA+OA, and (vii) SREBP‐1c siRNA+OA+EPA. *p < .05 versus controls. ^#^ *p* < .05 versus OA alone treatment. The data are expressed as the mean ± *SEM*. *n* = 6 in each treatment. EPA, eicosapentaenoic acid; OA, oleic acid; SREBP‐1c, sterol regulatory element‐binding protein‐c; TG, triglyceride

Oil Red O staining revealed small lipid droplets in normal HepG2 cells. However, intracellular lipid droplets were increased in OA‐treated cells, which were then reduced following EPA treatment for 24 hr. The lipid accumulation observed in HepG2 cells treated with OA was abolished by treatment with SREBP‐1c siRNA (*p* < .05; Figure 8A). The TG content was consistent with the accumulation of lipid droplets (*p* < .05; Figure 8B).

### 3.9 ACC expression in response to EPA or SREBP‐1c siRNA in HepG2 cells

**FIGURE 9:**
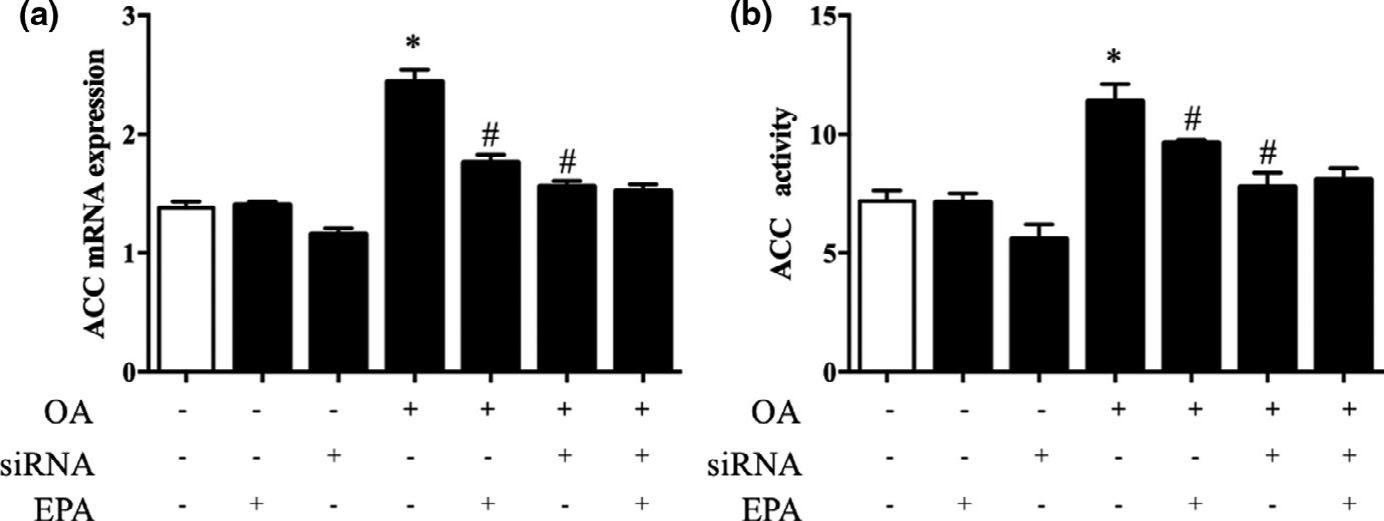
Lipogenesis in HepG2 cells with EPA treatment. (A) ACC mRNA expression and (B) ACC enzyme activity in HepG2 cells with different treatments: (i) Control, (ii) EPA, (iii) SREBP‐1c siRNA, (iv) OA, (v) OA+EPA, (vi) SREBP‐1c siRNA+OA, and (vii) SREBP‐1c siRNA+OA+EPA. *p < .05 versus controls, ^#^ *p* < .05 versus OA treatment alone. The data are expressed as the mean ± *SEM*. *n* = 6 per treatment. ACC, acetyl‐CoA carboxylase; EPA, eicosapentaenoic acid; OA, oleic acid; SREBP‐1c, sterol regulatory element‐binding protein‐c

In order to determine whether the regulation of EPA on ACC was mediated by SREBP‐1c, we next examined the mRNA expression of ACC. As expected, the ACC expression was increased by OA and further inhibited by EPA (*p* < .05; Figure 9A). SREBP‐1c siRNA partially prevented the OA‐induced overexpression of ACC, and EPA treatment could not decrease the ACC expression further (*p* < .05; Figure 9A). Similar to the ACC mRNA expression, the ACC enzyme activity was higher in cells treated with OA, but lower in those stimulated with EPA or treated with SREBP‐1c siRNA (*p* < .05; Figure 9B).

## 4 DISCUSSION

Epidemiological and clinical data indicate that postnatal overfeeding is strongly associated with a higher risk of developing obesity and metabolic syndrome later in life (Gluckman et al., 2008; McGill, 1998). Reducing litter size to induce postnatal overfeeding has been widely used to study major short‐ and long‐term outcomes of childhood obesity and related metabolic disease. Consistent with previous studies (Costa et al., 2019; Li et al., 2016; Yzydorczyk et al., 2019), our study demonstrated that postnatal overfed induced persistent increases in body weight, abnormal glucose, and hepatic steatosis in adulthood. The most important finding was that postweaning ω3PUFAs diet prevented the weight gain and reduced the risk of NAFLD development by SL rearing. Moreover, ω3PUFAs inhibited hepatic lipid accumulation by down‐regulating ACC and SREBP‐1c.

Numerous studies have demonstrated that ω3PUFAs are beneficial for NAFLD by reducing intrahepatic triglyceride contents and improving insulin sensitivity in human adults and animals (Alwayn et al., 2005; Levy et al., 2004; Svegliati‐Baroni et al., 2006). Low consumption of ω3PUFAs is risk factors for pediatric NAFLD development (St‐Jules et al., 2013). However, whether ω3PUFAs supplementation can prevent pediatric NAFLD remains controversial. The beneficial effects of ω3PUFAs supplementation on NAFLD depend on different dose and time (Mann et al., 2019). In our previous study (Dai et al., 2016), ω3PUFAs diet from postweaning, but not puberty around, is the most effective for SL‐induced obesity. Here, we observed that ω3PUFAs diet had a preventive effect on hepatic lipid accumulation in SL rats: decreased liver weights, hepatic TG contents, and NAS scores (with mild or even no macrovesicular steatosis). Notably, food intake did not decrease in SL rats, which indicated that ω3PUFAs could improve metabolic programming effects on hepatic lipid accumulation rather than via the restriction of energy. In addition, we did not identify changes in hepatic glucose and hepatic lipid metabolism in NL‐FO rats, suggesting that the effectiveness of ω3PUFAs (at this concentration) might be in the context of metabolic disorders.

Increasing evidence has verified that ω3PUFAs can prevent NAFLD by regulating hepatic lipid metabolism (Dentin et al., 2005; Pawar & Jump, 2003). Specifically, ω3PUFAs are capable of inhibiting hepatic FASN, ACC, and SREBP‐1c expression involved in fatty acid synthesis (Jump, 2008) and enhancing lipid oxidation via PPARα pathway (Lefils et al., 2010). In postnatal overfed rats, the metabolic programming effects of hepatic lipogenesis could be mainly involved in DNL (Ji et al., 2014). Therefore, in this study, we focused on DNL and confirmed that postweaning ω3PUFAs markedly recovered ACC gene expression and activity to normal levels. In contrast, the effects of ω3PUFAs on LPL, L‐FABP, SCD1, and CPT1 are relatively weak. However, the effect of ω3PUFAs on MTP expression was not obvious, which suggested that other factors (such as insulin, Chan et al., 2015; or inflammatory factors, Saraswathi et al., 2013) could be responsible for the regulation of lipid export. All such data indicated that ω3PUFAs intervention could prevent hepatic steatosis by suppressing hepatic lipid synthesis, especially revising the ACC expression pattern induced by postnatal overfeeding.

Based on these findings, we further explored the mechanism of ω3PUFAs on hepatic lipid metabolism in HepG2 cells. ω3PUFAs are composed of EPA, docosahexenoic acid (DHA), and alpha linolenic acid (ALA). The effects of ALA on hepatic lipid metabolism are weak. EPA is more widely used in in vitro studies of hepatic lipid metabolism (Nanthirudjanar et al., 2013; Notarnicola et al., 2010), while DHA is shown to be more effective at suppressing hepatic inflammation, fibrosis, and oxidative stress in animal model (Jump et al., 2013). Here, we confirmed that EPA stimulation (at 50 µmol/L) in HepG2 cells using OA treatment had a significant inhibitory effect on hepatic lipid accumulation and ACC gene expression. However, normal cells were unaffected by EPA treatment, which suggested that EPA primarily affected those lipids accumulated in hepatic cells.

SREBP‐1c is thought to play a significant role in hepatic DNL signaling. Normally, hepatic SREBP‐1c expression is maintained at a low levels and could directly regulate the enzymes involved in glucose and lipid metabolism, including ACC, FASN, and SCD1. However, SREBP‐1c expression could be easily affected by different nutritional patterns (Deng et al., 2015; Jump, 2008; Jump et al., 2005). High fat diet could increase hepatic SREBP‐1c expression, but ω3PUFAs decrease hepatic SREBP‐1c and liver X receptor expression. At present study, we found that postweaning ω3PUFAs decreased SREBP‐1c expression, which coincided with ACC expression in postnatal overfed rats. This synergic relationship was further confirmed in vitro using HepG2 cells. All such observations indicated that SREBP‐1c could be mediated via ω3PUFAs regulation of ACC. Furthermore, we confirmed that when SREPB‐1c was knocked‐down first, EPA treatment failed to display any action on ACC, thus indicating that EPA affected ACC expression on the basis of SREBP‐1c.

In conclusion, postweaning dietary with fish oil could improve hepatic steatosis and decrease the risk of developing NAFLD. More importantly, ω3PUFAs reduced hepatic lipid accumulation by down‐regulating ACC activity through the SREBP‐1c pathway in rats. However, there were some limitations to this study. First, our study performed to date suggests that ω3PUFAs supplementation maybe effective in the early stages of NAFLD, and further studies are needed to evaluate the efficacy of ω3PUFAs diet on severe NAFLD or even NASH. Second, we found ω3PUFAs can prevent NAFLD of postnatal overfed rats via regulating other lipid metabolic enzymes, such as FASN, SCD1, LPL, and CPT1; thus, further in vitro study should be evaluated. Third, DHA, as another important component of ω3PUFAs, should also be concerned in postnatal overfeeding. Recently, a review article suggested that ω3PUFAs might be a safe and effective intervention in both children and adults (Spooner & Jump, 2019). Therefore, our finding emphasizes that the ω3PUFAs supplementation should be encouraged in obese children, especially those who have experienced postnatal overfed.

## CONFLICT OF INTEREST

None.

## AUTHOR CONTRIBUTIONS

**Nan Zhou:** Data curation (lead); Methodology (lead); Project administration (lead); Writing‐original draft (lead); Writing‐review & editing (lead). **susu Du:** Data curation (supporting); Methodology (supporting). **yanyan Dai:** Methodology (supporting). **fan Yang:** Methodology (supporting). **xiaonan Li:** Project administration (lead); Writing‐original draft (supporting); Writing‐review & editing (lead).

## ETHICAL APPROVAL

This study was approved by Unit for Laboratory Animal Medicine at Nanjing Medical University.
